# Altered functional connectivity of the primary motor cortex in tremor dominant and postural instability gait difficulty subtypes of early drug-naive Parkinson’s disease patients

**DOI:** 10.3389/fneur.2023.1151775

**Published:** 2023-05-12

**Authors:** Qi Wang, Miao Yu, Lei Yan, Jianxia Xu, Yajie Wang, Gaiyan Zhou, Weiguo Liu

**Affiliations:** ^1^Department of Neurology, Affiliated Brain Hospital of Nanjing Medical University, Nanjing, China; ^2^Department of Neurology, Affiliated Hospital of Jiangsu University, Zhenjiang, China

**Keywords:** Parkinson’s disease, primary motor cortex, tremor dominant, postural instability and gait difficulty, resting-state functional connectivity

## Abstract

**Background:**

The primary motor cortex (M1) is an important hub in the motor circuitry of Parkinson’s disease (PD), but the subregions’ function and their correlation to tremor dominant (TD) and postural instability and gait disturbance (PIGD) with PD remain unclear. This study aimed to determine whether the functional connectivity (FC) of the M1 subregions varied between the PD and PIGD subtypes.

**Methods:**

We recruited 28 TD patients, 49 PIGD patients, and 42 healthy controls (HCs). M1 was divided into 12 regions of interest using the Human Brainnetome Atlas template to compare FC among these groups.

**Results:**

Compared with HCs, TD and PIGD patients exhibited increased FC between the left upper limb region (A4UL_L) and the right caudate nucleus (CAU)/left putamen (PUT), between the right A4UL (A4UL_R) and the left anterior cingulate and paracingulate gyri (ACG)/bilateral cerebellum4_5 (CRBL4_5)/left PUT/right CAU/left supramarginal gyrus/left middle frontal gyrus (MFG), as well as decreased connectivity between the A4UL_L and the left postcentral gyrus and the bilateral cuneus, and between the A4UL_R and the right inferior occipital gyrus. TD patients showed increased FC between the right caudal dorsolateral area 6 (A6CDL_R) and the left ACG/right MFG, between the A4UL_L and the right CRBL6/right middle frontal gyrus, orbital part/bilateral inferior frontal gyrus, and orbital part (ORBinf), and between the A4UL_R and the left ORBinf/right MFG/right insula (INS). PIGD patients displayed increased connectivity between the A4UL_L and the left CRBL4_5. Compared with PIGD patients, TD patients exhibited increased connectivity between the A6CDL_R and the left ACG/right MFG and between the A4UL_R and the left ACG/left ORBinf/right INS/right MFG. Furthermore, in TD and PIGD groups, the FC strength between the A6CDL_R and right MFG was negatively correlated with PIGD scores, while the FC strength between the A4UL_R and left ORBinf/right INS was positively correlated with TD scores and tremor scores.

**Conclusion:**

Our results demonstrated that early TD and PIGD patients share some common injury and compensatory mechanisms. TD patients occupied more resources in the MFG, ORBinf, INS, and ACG, which can be used as biomarkers to distinguish them from PIGD patients.

## Introduction

Parkinson’s disease (PD) is a neurodegenerative disorder characterized by a variable combination of slow motor rigidity and 4–6 Hz resting tremor, with gait and postural reflex disturbances occurring in a fair proportion of patients (1). A growing number of scholars have proposed that heterogeneous motor symptoms in early Parkinson’s disease can be conceptualized by clinical subtypes, including the internationally recognized tremor dominant (TD)/intermediate/postural instability gait difficulty (PIGD) subtypes classification proposed by Jankovic in 1990 (2). Since the initial characterization of motor subtypes, clinical research on PD has been developed in many aspects, including the role of an increasing number of neuroimaging markers in the diagnosis and typing of PD. Previous studies have found that compared with TD patients, PIGD patients are more susceptible to depressive symptoms, poorer cognitive function, more rapid disease progression, and a worse quality of life (3–6), which may be related to the underlying differences in neuropathological mechanisms between the two motor subtypes.

The fundamental pathophysiological mechanism of PD is the degeneration of dopaminergic neurons, resulting in the loss of dopamine in the striatum. This loss leads to dysfunction of the basal ganglia-thalamus-motor cortex (BGMC) circuit. The primary motor cortex (M1) is an important node in this motor circuit, playing a core role in the production of nerve impulses that control movement (7, 8). Bologna et al. (9) discovered that bradykinesia in patients with Parkinson’s disease was associated with neurophysiological abnormalities in M1, while additional processes that are dopamine-sensitive must also be involved. According to one study, M1 plays a significant role in the pathophysiological mechanisms that underlie rest tremor and re-emergent tremor in PD patients (10). A further study found that PD patients with TD had increased functional connectivity (FC) between the thalamus, M1, and intermediate nucleus of the thalamus (VIM) and that their FC values were positively correlated with the resting tremor score (11). These investigations demonstrate a strong correlation between M1 functional activity and clinical motor symptoms in PD patients. However, there are also controversial aspects of the M1 research in PD-related imaging studies. For example, a PD study in 2009 showed increased ReHo in cerebellar and M1 regions and that the increased ReHo could be normalized by levodopa medication (12). In 2017, a study reached the opposite conclusion, with PD patients showing reduced ReHo in M1 and basal ganglia areas and further reductions as the disease progressed (13, 14). The reason for this difference in results may be due to differences in age, number, disease duration, and main symptoms of the patients enrolled. It also illustrates that the M1 region is complex and extensive, encompassing different internal structural functions. The functional activities of each subregion within M1 are not described in any of the aforementioned research, which instead focused on M1 as a whole. M1 has multiple internal structural roles, and its sub-regions are functionally directly related to their counterparts in the contralateral motor cortex on a one-to-one basis (15). Given the close association of M1 with PD motor symptoms, as a key node of the BGMC loop and the motor cortex-cerebellar-thalamic loop, it is worth investigating whether M1 subregions show characteristic functional connectivity changes in PD patients with the motor subtype.

Resting-state functional magnetic resonance imaging (RS-fMRI) is known as a non-invasive technique that does not involve any task-based activities. A growing number of fMRI studies have recently begun to look into resting-state functional connectivity (RSFC) in Parkinson’s disease. M1 is crucial for motor execution, and the pattern of RSFC in this area is altered in PD patients (16). Changes in M1 functional connectivity are associated with dopamine depletion in the putamen in PD patients and investigating the patterns of these changes may help to elucidate the underlying pathophysiological mechanism of Parkinson’s disease (17). In the current study, M1 was divided into 12 subregions using the Brainnetome Atlas template, and the FC variations in each subregion in PD patients with TD and PIGD subtypes were assessed. In contrast to the conventional AAL template, the Brainnetome Atlas template uses differences in each voxel’s structural connection pattern [diffusion tensor imaging (DTI) for fiber tracking] and aggregates the voxels with similar connection patterns using a clustering algorithm to define the boundaries of each brain region (18). This template has given us a more thorough grasp of the FC characteristics in brain area subregions, such as the Broca’s area (19) and thalamus (20). Considering that previous studies have demonstrated that anti-parkinsonian therapy, such as levodopa, causes connectivity changes (21), we focused on drug-naive PD patients with TD and PIGD subtypes to eliminate the influence of medication. We hypothesized that (1) PD patients with TD and PIGD subtypes would show different FC of M1 subregions and (2) altered FC of M1 subregions may account for their clinical features.

## Materials and methods

### Participants

The Affiliated Brain Hospital of Nanjing Medical University’s Medical Ethics Committee approved the current experiment, and each participant gave their informed consent in writing. A total of 97 PD patients and 45 healthy controls (HCs) were recruited from the Department of Neurology of the Affiliated Brain Hospital of Nanjing Medical University between October 2018 and October 2020. According to the United Kingdom Parkinson’s Disease Society Brain Bank criteria (22), an experienced movement disorder specialist made a diagnosis of Parkinson’s disease for each subject. To exclude dementia and severe brain atrophy, structural brain MRIs were performed on all PD patients and HCs. The requirements for all subjects were as follows: subjects who (1) are right-handed, (2) were aged 40–80 years, and (3) were sighted or with corrected sighted and binaural hearing, meeting the assessment requirements and completing the examination. The inclusion criteria for PD patients were as follows: (1) the diagnostic criteria for PD met the Kingdom Parkinson Disease Society Brain Bank Criteria for idiopathic PD, (2) *de novo* PD patients without medication, and (3) a Mini-Mental State Examination (MMSE) score of ≥24. The following were the exclusion criteria for all subjects: subjects with (1) a history of impaired consciousness; (2) a history of manic episodes, schizophrenia, or other psychiatric diseases; (3) a history of addiction to alcohol or drugs; (4) complications of severe brain, heart, kidney, liver, and hemopoietic system diseases; (5) contraindications to MRI scanning such as implantation of electronic and metallic devices; and (6) T2-weighted MRI showing vascular damage or cerebral infarction.

Clinical and imaging data were obtained before the initiation of any treatment. Clinical symptoms were assessed using rating scales before the MRI scan. The severity of depression and anxiety was quantified using the 24-item Hamilton Depression Rating Scale (HAMD) and the 14-item Hamilton Anxiety Scale (HAMA). Cognitive function was evaluated with the Montreal Cognitive Assessment (MoCA) and Mini-Mental State Examination (MMSE). The PD severity was evaluated by using the unified PD rating scale (UPDRS) and the Hoehn and Yahr stage (H-Y). The classification of TD and PIGD subtypes was based on the approach adopted by Jankovic et al. in 1990 (2). Using UPDRS II and III, the ratio of the mean UPDRS tremor scores (UPDRS II item 16 and UPDRS III items 20–21 divided by 8) to the mean UPDRS PIGD scores (UPDRS II items 13–15 and UPDRS III items 29–30 divided by 5) was used to identify TD (ratio ≥ 1.5 or PIGD score = 0 and TD score > 0), indeterminate (1.0 < ratios <1.5 or both TD and PIGD scores = 0), and PIGD (ratio ≤ 1 or TD score = 0 and PIGD score > 0) PD patients. A total of 15 intermediate patients, two TD patients with poor MR image quality, three PIGD patients, and three HCs with large head movements were excluded. Finally, 28 TD patients (male 16/ female 12), 49 PIGD patients (male 24/ female 25), and 42 HC patients (male 20/ female 22) who matched for age, sex, and education were enrolled. The demographics and clinical details are shown in Table 1.

**Table 1:** Demographic and clinical characteristics of the participants (mean ± SD).

Variable	TD (28)	PIGD (49)	HC (42)	*P*-value
Age (years)	61.14 ± 6.59	59.02 ± 7.84	59.83 ± 5.20	0.414^a^
Gender (M/F)	16/12	24/25	20/22	0.712^c^
Education (years)	9.04 ± 4.83	10.20 ± 3.30	11.07 ± 3.16	0.160^b^
Disease duration (months)	24.43 ± 15.28	21.39 ± 15.40	/	0.324^e^
H-Y stage	1.71 ± 0.46	1.536 ± 0.52	/	0.151^e^
UPDRS II	7.86 ± 3.92	8.06 ± 3.54	/	0.201^d^
UPDRS III	25.18 ± 12.73	24.82 ± 11.74	/	0.692^d^
Tremor	5.75 ± 3.92	1.71 ± 1.54	/	<0.001^e^
Rigidity	5.68 ± 4.21	5.94 ± 3.98	/	0.742^e^
Bradykinesia	10.39 ± 6.09	12.61 ± 7.09	/	0.470^d^
Axial symptoms	2.11 ± 1.42	3.31 ± 1.90	/	0.008^e^
TD scores	7.71 ± 4.32	2.65 ± 1.81	/	<0.001^e^
PIGD scores	1.79 ± 1.23	3.22 ± 1.18	/	<0.001^e^
MMSE	27.39 ± 1.95 ^g**^	27.41 ± 1.91 ^h***^	28.79 ± 1.40	<0.001^b^
MoCA	21.54 ± 4.32 ^g***^	22.10 ± 3.91 ^h***^	24.86 ± 3.12	<0.001^b^
HAMD	8.11 ± 5.38 ^g**^	11.73 ± 8.51 ^h***^	3.79 ± 4.74	<0.001^b^
HAMA	4.89 ± 4.06	7.71 ± 5.43 ^h***^	2.86 ± 4.18	<0.001^b^

UPDRS, Unified Parkinson’s Disease Rating Scale; H-Y stage, Hoehn and Yahr stage; TD, Tremor Dominant; PIGD, postural instability/gait difficulty; MMSE, Mini-Mental State Examination; MoCA, Montreal Cognitive Assessment; HAMD, Hamilton Depression Rating Scale; HAMA, Hamilton Anxiety Rating Scale. ^a^One-way analysis of variance (ANOVA) tests; ^b^Kruskal–Wallis H-test; ^c^chi-square test; ^d^two-sample t-test; ^e^Mann–Whitney U-*t*est; ^f^PD-TD vs. PD-PIGD; ^g^PD-TD vs. HC; ^h^PD-PIGD vs. HC. ^*^p < 0.05; ^**^p < 0.01, ^***^p < 0.001. UPDRS III was divided into subscores for tremor (UPDRS III items 20 and 21), rigidity (UPDRS III item 22), bradykinesia (UPDRS III items 23–26 and 31), and axial symptoms (UPDRS III items 27–30) (23). TD scores (UPDRS II item 16 and UPDRS III item 20, 21); PIGD scores (UPDRS II item 13–15 and UPDRS III item 29, 30) (2).

### Functional magnetic resonance imaging procedure

MRI was performed using a 3 T MRI scanner (Siemens, Verio, Germany). All participants were placed in the supine position with their heads immobilized by foam pads with a normal birdcage head coil to reduce head movement. The subjects were requested to remain as motionless as possible and to keep their eyes closed while staying awake and not thinking about anything. Axial anatomical images were acquired using a T1 fluid-attenuated inversion recovery sequence with parameters as follows: repetition time (TR) = 2,530 ms; echo time (TE) = 3.34 ms; field of view (FOV) = 256 × 256 mm^2^; voxel sizes = 1.0 × 1.0 × 1.3 mm^3^; matrix = 256 × 192; slice thickness/gap = 1.33/0.5 mm; flip angle (FA) = 7 degrees; and bandwidth = 180 HZ/PX; 128 slices covered the whole brain for image registration and functional localization. Functional images were subsequently collected in the same slice orientation with a gradient-recalled echo-planar imaging pulse sequence, which included 240 volumes. The parameters were as follows: TR = 2,000 ms; TE = 30 ms; FOV = 220 × 220 mm^2^; voxel sizes = 3.44 × 3.44 × 4.13 mm^3^; matrix = 64 × 64; thickness/gap = 3.5/0.63 mm; FA = 90 degrees; bandwidth = 2,232 HZ/PX; and slice numbers = 31.

### Image preprocessing

The toolbox for data processing and analysis for (resting-state) brain imaging (DPABI 5.1)^1^ based on the MATLAB 2013b platform was used to preprocess the fMRI data. The preprocessing steps are as follows: the initial 10 volumes of the blood oxygenation level-dependent data were eliminated to allow for participant acclimatization to the scanning environment and signal balance. The remaining images were corrected for slice timing using the middle slice as a reference so that the image acquisition time at all levels of the whole brain can be consistent. Subsequently, the time series of each subject underwent realignment correction to account for head motion. A total of six subjects with head motions exceeding 3.0 mm translation or 3.0° rotation were excluded. Thereafter, the functional images were co-registered to the corresponding T1 images, which were further segmented into gray matter, white matter, and cerebrospinal fluid (CSF) using a unified segmentation method.^2^ Finally, the functional images were resampled to a 3 × 3 × 3 mm^3^ voxel size and normalized into the standard Montreal Neurological Institute space by the Diffeomorphic Anatomical Registration Through Exponentiated Lie (DARTEL) algebra. Furthermore, several nuisance variables, including the Friston-24 motion parameters (six head motion parameters, six head motion parameters one-time point earlier, and the 12 corresponding squared items) (24), CSF and white matter signals, and the linear and quadratic trends, were regressed out to minimize the motion artifact and improve the signal-noise ratio. The resultant fMRI data were bandpass filtered (0.01 < *f* < 0.1 Hz), and spatial smoothing was performed with a 6-mm full width at half-maximum (FWHM) Gaussian kernel.

### Functional connectivity analysis

A total of 12 M1 subregions from the Human Brainnetome Atlas^3^ were adopted for this research, including the bilateral head and face region, bilateral caudal dorsolateral area 6 (A6CDL), bilateral upper limb region (A4UL), bilateral trunk region, bilateral tongue, larynx region, and bilateral caudal ventrolateral area 6 as the ROIs. The mean time series were separately extracted for each ROI. Subsequently, FC analysis was performed by computing the temporal Pearson’s correlation between the mean time series of each ROI and the time series of each voxel within the brain. Fisher’s r-to-z transformation converted the resulting connectivity maps to Z maps to improve normality, thereby creating a Z-score map for each ROI per subject.

### Statistical analysis

We utilized the SPSS 25.0 software for demographic statistical analysis (IBM, United States). To compare the TD, PIGD, and HCs groups, one-way analyzes of variance (ANOVA) or the Kruskal–Wallis H-test was used. The Mann–Whitney U-test or two-sample *t*-test was used to compare the TD and PIGD groups. Using the chi-squared test, categorical data including gender were compared. We used the DPABI statistics modules for the statistical analysis of the fMRI data. Gray matter volumes were calculated in the step of T1 image segmentation and used as covariates to eliminate the influences of gray matter in our senile subjects. With gray matter volume, age, gender, and education as covariates, we used the ANCOVA to determine the differences in FC among TD patients, PIGD patients, and HCs for each ROI. *Post hoc* two-sample *t*-tests were performed. The false discovery rate (FDR) correction with a value of *p* of < 0.05 was used to do multiple comparison corrections in the FC study. Spearman correlation analysis was performed to assess the correlation between the FC values in the brain regions with group difference and clinical measures (including UPDRS II, UPDRS-III, tremor, rigidity, bradykinesia, axial symptom, TD, PIGD, disease duration, MMSE, MoCA, HAMD, and HAMA scores) in patients with PD (*p* < 0.05, Bonferroni corrected).

SPSS v25 software was used for binary logistic regression analysis to test the diagnostic value of the FC values in the brain regions with group differences in patients with TD and PIGD. FC indicators in univariate analysis were incorporated into the multi-factor model and were eliminated backward according to the likelihood ratio. The variable was selected with a criterion of a value of *p* of < 0.05. We then estimated the receiver operating characteristic curve (ROC) and area under the curve (AUC) to evaluate the predictive power, including accuracy, sensitivity, and specificity, of univariate and multivariate analysis models.

## Results

### Demographic and clinical characteristics

As shown in Table 1, there were no significant differences in disease duration, H-Y stage, UPDRS II, UPDRS-III, rigidity, and bradykinesia (*P*>0.05) between the TD scores and PIGD patients, while no significant differences in age, gender, and education level (*P*>0.05) among the three groups were observed. In contrast, significant variations in the tremor, axial symptoms, TD scores, and PIGD scores were observed between the TD and PIGD groups (*p* < 0.05), and significant differences in the MMSE, MoCA, HAMD, and HAMA were observed among the three groups (*p* < 0.001).

### Functional connectivity

**Figure 1:**
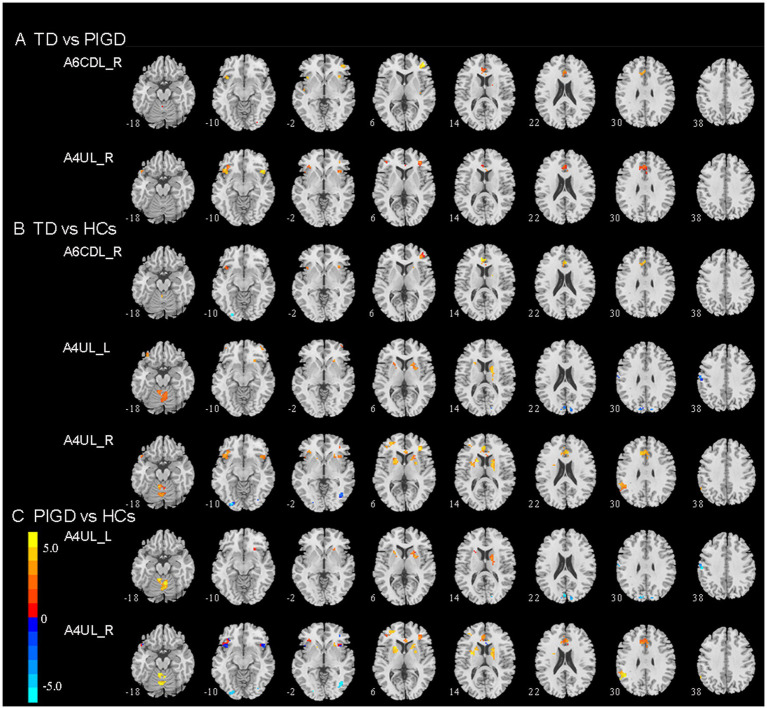
Brain region exhibiting a significant difference in FC between **(A)** TD and PIGD patients; **(B)** TD patients and HCs; **(C)** PIGD patients and HCs (*p* < 0.05, FDR corrected). TD, tremor dominant; PIGD, postural instability and gait dysfunction; HCs, healthy controls; A6cdl, caudal dorsolateral area 6; A4ul, upper limb region.

We mainly observed the difference in FC on the motor circuit node of M1 subregions among the three groups (Table 2; Figure 1). These subregions of M1 and their specifics are as follows.

**Table 2:** Changes in functional connectivity between groups.

Seed ROI	Brain region(AAL)	L/R	Peak MNI coordinates *x y z*			Cluster size (voxels)	*t*-value
TD vs. PIGD							
A6CDL_R	ACG	L	−3	30	27	102	4.461
	MFG	R	42	45	3	50	5.2006
A4UL_R	ACG	L	0	33	27	130	4.2838
	ORBinf	L	−39	18	−12	67	4.6371
	INS	R	45	18	−9	36	4.9901
	MFG	R	36	36	0	33	4.1243
TD vs. HCs							
A6CDL_R	ACG	L	−6	36	15	102	5.2269
	MFG	R	39	39	9	37	4.3804
A4UL_L	CRBL6	R	27	−69	−27	253	5.637
	CAU	R	15	3	18	74	5.021
	PoCG	L	−54	−24	45	37	−4.3224
	CUN	R	15	−84	18	32	−3.6801
	ORBinf	R	24	24	−9	29	4.3557
	PUT	L	−24	18	12	27	4.7621
	CUN	L	−3	−84	24	26	−4.3224
	ORBmid	R	39	51	−15	20	4.0113
	ORBinf	L	−36	36	−18	20	4.234
A4UL_R	ACG	L	−3	39	15	171	4.9123
	CRBL4_5	L	−9	−48	−18	127	4.3914
	PUT	L	−21	9	12	98	5.063
	CAU	R	18	3	18	88	5.3846
	ORBinf	L	−42	21	−9	67	4.2524
	SMG	L	−57	−42	30	57	4.4747
	MFG	L	−30	48	6	49	4.5388
	MFG	R	36	36	6	40	5.2594
	INS	R	42	18	−6	36	4.0216
	IOG	R	39	−66	0	25	−3.0237
	CRBL4_5	R	24	−36	−33	23	5.264
PIGD vs. HCs							
A4UL_L	CRBL4_5	L	−9	−48	−18	252	5.5179
	CAU	R	24	6	6	72	4.4041
	PoCG	L	−54	−21	39	38	−4.3203
	CUN	R	15	−87	18	32	−3.9912
	CUN	L	−3	−81	24	28	−4.3376
	PUT	L	−21	15	6	26	4.4351
A4UL_R	CRBL4_5	L	−6	−48	−18	127	4.5518
	PUT	L	−30	0	18	98	4.7621
	CAU	R	18	12	12	88	4.1164
	SMG	L	−48	−45	33	57	4.5254
	MFG	L	−27	48	3	43	4.0271
	IOG	R	39	−66	−3	29	−5.1367
	CRBL4_5	R	27	−39	−30	23	3.7693
	ACG	L	−9	24	21	20	2.973

ROI, region of interest; AAL, anatomical automatic labeling; L/R, left/right; MNI, Montreal Neurological Institute; TD, tremor dominant; PIGD, postural instability and gait dysfunction; HCs, healthy controls; A6cdl, caudal dorsolateral area 6; A4ul, upper limb region; ACG, anterior cingulate and paracingulate gyri; MFG, middle frontal gyrus; ORBinf, inferior frontal gyrus, orbital part; INS, insula; CRBL6, cerebellum 6; CAU, caudate nucleus; PoCG, postcentral gyrus; CUN, cuneus; PUT, putamen; ORBmid, middle frontal gyrus, orbital part; CRBL4_5, cerebellum 4_5; SMG, supramarginal gyrus; IOG, inferior occipital gyrus. The results represent a comparison of functional connectivity in ROIs between TD and PIGD, between TD and HCs, and between PIGD and HCs (*p* < 0.05, FDR corrected, cluster size > 19 voxels).

### Tremor dominant vs. PIGD

Compared with the PIGD patients, the TD patients showed increased connectivity between the right A6CDL (A6CDL_R) and the left anterior cingulate and paracingulate gyri (ACG) and the right middle frontal gyrus (MFG) and between the right A4UL (A4UL_R) and the left ACG/ left inferior frontal gyrus and orbital part (ORBinf)/right insula (INS)/right MFG (Figure 1A; *p* < 0.05, FDR corrected).

### Tremor dominant vs. HCs

Compared with the HCs, the TD patients showed increased connectivity between the A6CDL_R and the left ACG and the right MFG; between the left A4UL (A4UL_L) and the right cerebellum6 (CRBL6)/right caudate nucleus (CAU)/bilateral ORBinf/left putamen (PUT)/right middle frontal gyrus, and orbital part (ORBmid); between the A4UL_R and the left ACG/bilateral CRBL4_5/left PUT/right CAU/left ORBinf/left supramarginal gyrus (SMG)/bilateral MFG/right INS, as well as decreased connectivity between the A4UL_L and the left postcentral gyrus (PoCG) and the bilateral cuneus (CUN), and between the A4UL_R and the right inferior occipital gyrus (IOG) (Figure 1B; *p* < 0.05, FDR corrected).

### Postural instability and gait disturbance vs. HCs

Compared with the HCs, the TD patients exhibited increased connectivity between the A4UL_L and the right CRBL4_5/right CAU/left PUT, between the A4UL_R and the bilateral CRBL4_5/left PUT/right CAU/ left SMG/left MFG/left ACG, as well as decreased connectivity between the A4UL_L and the left PoCG and the bilateral CUN, and between the A4UL_R and the right IOG (Figure 1B; *p* < 0.05, FDR corrected).

### Correlation analysis

**Figure 2:**
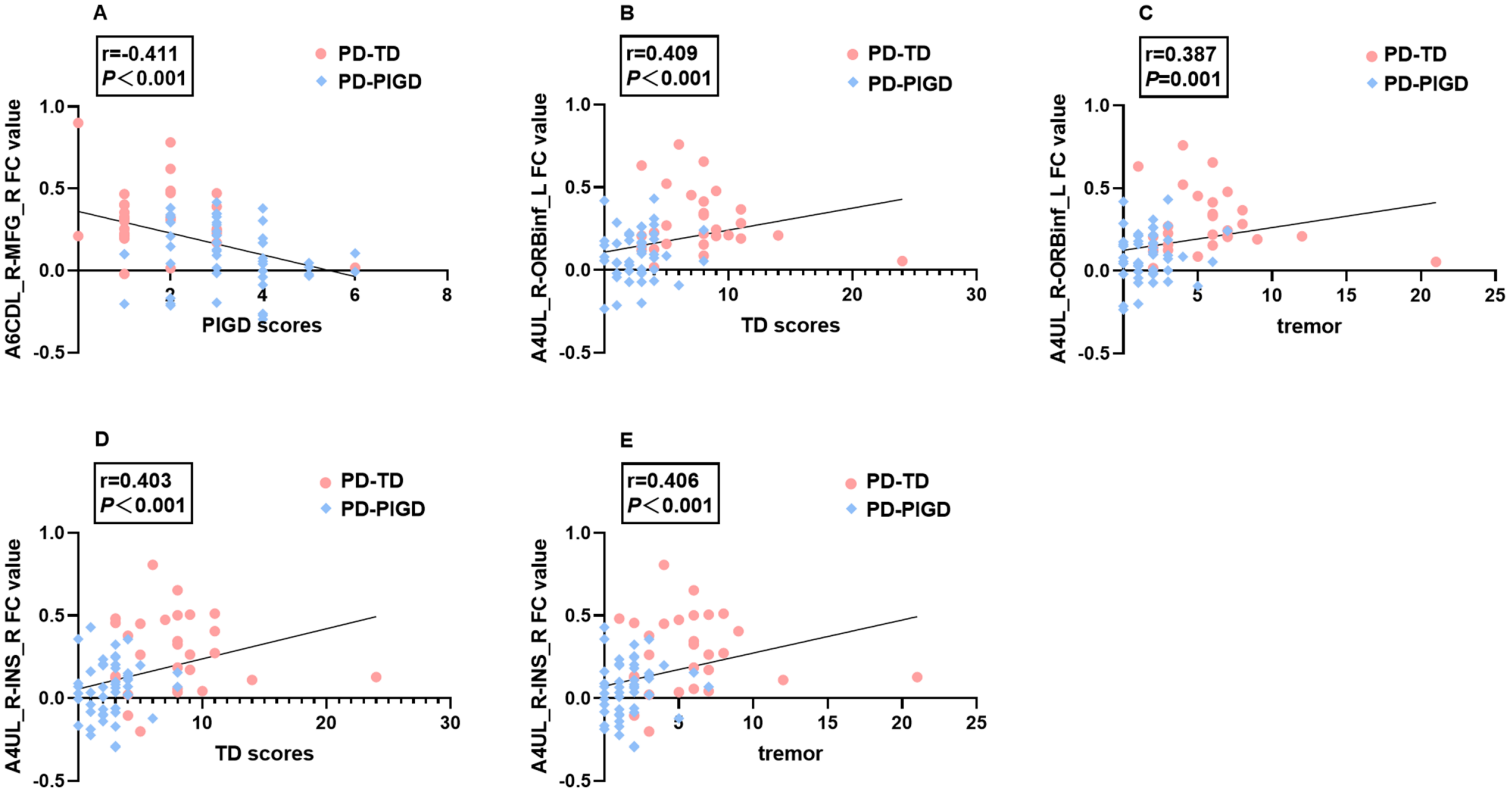
Significant associations between altered FC values and tremor, TD, and PIGD scores (*p* < 0.05, Bonferroni corrected). A6CDL_R-MFG_R FC value negatively correlated with PIGD scores **(A)**. A4UL_R-ORBinf_L FC value positively correlated with TD scores **(B)** and tremor scores **(C)**. A4UL_R-INS_R FC value positively correlated with TD scores **(D)** and tremor scores **(E)**.

The Spearman correlation analysis demonstrated that in TD and PIGD groups, the FC values between the A6CDL_R and right MFG were negatively correlated with PIGD scores (*r* = −0.411, *p* < 0.001), while the FC values between the A4UL_R and left ORBinf were positively correlated with TD scores (*r* = 0.409, *p* < 0.001) and tremor scores (*r* = 0.387, *p* = 0.001), and the FC values between the A4UL_R and right INS were positively correlated with TD scores (*r* = 0.403, *p* < 0.001) and tremor scores (*r* = 0.406, *p* < 0.001; Figure 2).

### Classification of TD and PIGD subtypes using the ROC analysis

The altered FC value to distinguish the TD and PIGD subtypes was assessed using a ROC analysis. In the TD and PIGD groups, the AUC value of FC between A6CDL_R and MFG_R was 0.798, with a *p*-value of <0.001 with sensitivity = 89.3% and specificity = 63.3%. The AUC value of FC between A4UL_R and ORBinf_L was 0.781, with a *p*-value of <0.001 with sensitivity = 71.4% and specificity = 73.5%. In the TD and HCs groups, the AUC value of FC between A6CDL_R and ACG_L was 0.809, with a *p*-value of <0.001 with sensitivity = 85.7% and specificity = 64.3%. The AUC value of FC between A4UL_L and CRBL6_R was 0.810, with a *p*-value of <0.001 with sensitivity = 67.9% and specificity = 83.3%. The AUC value of FC between A4UL_L and PoCG_L was 0.739, with a *p*-value of 0.001 with sensitivity = 61.9% and specificity = 85.7%. The AUC value of FC between A4UL_R and ORBinf_L was 0.810, with a *p*-value of <0.001 with sensitivity = 78.6% and specificity = 76.2%. In the PIGD and HCs groups, the AUC value of FC between A4UL_L and CRBL4_5_L was 0.793 with a *p*-value of <0.001 with sensitivity = 65.3% and specificity = 83.3%. The AUC value of FC between A4UL_L and CUN_R was 0.714, with a *p*-value of 0.001 with sensitivity = 47.6% and specificity = 87.8%. The AUC value of FC between A4UL_R and SMG_L was 0.737, with a *p*-value of <0.001 with sensitivity = 87.8% and specificity = 61.9%.

**Figure 3:**
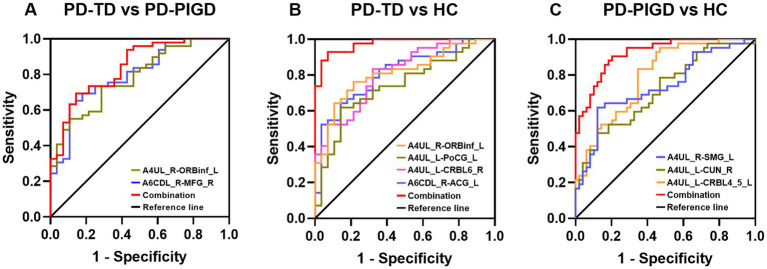
Diagnosis and differentiation of TD and PIGD based on ROC analysis. **(A)** ROC curve showing the classification of TD and PIGD. **(B)** ROC curve showing the classification of TD and HCs. **(C)** ROC curve showing the classification of PIGD and HCs.

As shown in Figure 3, the optimal classification model was based on the combination of multiple indicators. The AUC value of combined multiple indicators in the TD and PIGD groups was 0.840, with a *p*-value of <0.001 with sensitivity = 69.4% and specificity = 85.7%. In the TD and HCs groups, the AUC value of combined multiple indicators was 0.974 with a *p*-value of <0.001 with sensitivity = 92.9% and specificity = 92.9%. In the PIGD and HCs groups, the AUC value of combined multiple indicators was 0.922, with a *p*-value of <0.001 with sensitivity = 90.5% and specificity = 79.6%.

## Discussion

This study investigated the variation of FC patterns in M1 subregions of PD patients with TD and PIGD subtypes. We discovered that subregions of the M1, involving the right A6CDL and right A4UL, displayed significantly different changes in FC between TD and PIGD subtypes. Importantly, the altered FC was linked to certain clinical manifestations. First, the increased FC between the right A6CDL and right MFG was negatively correlated with PIGD scores. Second, the increased FC between the right A4UL and left ORBinf was positively related to TD scores and tremor scores. Third, the increased FC between the right A4UL and right INS was positively related to TD scores and tremor scores. Additionally, the combined multiple FC indicators of M1 subregions provided a potent biomarker for the classification of TD and PIGD. Our findings imply that PD patients with TD and PIGD subtypes exhibit distinct alterations in the M1 subregions. Multiple motor circuits or brain networks were affected by these changes in connection patterns, and they were also linked with certain clinical manifestations.

M1 is essential for producing the neuronal impulses that control movement (7). The motor cortex (MC) contains M1 and is involved in the MC–cerebellum–thalamus circuit, which may help to explain resting tremor (25), in addition to the conventional BGMC circuit, which is utilized to explain the underlying mechanism of bradykinesia and rigidity (9). Our study found differences in functional connectivity among the three groups with A4UL as the seed point in newly diagnosed drug-naïve PD patients, which is consistent with previous studies that motor symptoms in PD begin from one distal upper limb. A tremor in the upper extremities was the first symptom in 70% of individuals in a previous study of 672 PD patients conducted by Hoehn and Yahr (26). A study of a large cohort described 68% upper limb predominance in 1,244 PD patients (27). More recently, a study demonstrated that according to patients’ self-reports, neurologists’ impressions, and Movement Disorders Society Unified Parkinson’s Disease Rating Scale scores, 92% of patients in a newly diagnosed drug-naive PD cohort had the upper limbs as the most affected body region at the onset (28). Further analysis of this cohort revealed that the most severely affected side exhibited the greatest loss of 18F-DOPA uptake rate in the rostrocaudal and dorsoventral axes of the caudal and intermediate putamen, which corresponds to the somatic representation of the upper limb and is consistent with the onset of the upper limb clinically (29).

According to our findings, the second changed subregion of M1 was the right caudal dorsolateral area 6, the greatest differences of FC being from right A6CDL and bilateral A4UL involving ACG, MFG, INS, ORBinf, ORBmid, basal Ganges, CUN, PoCG, and SMG. According to a previous study, goal-directed motions are influenced by the frontal lobe, INS and parietal lobe, and ACG (30). As components of the executive control network (31), the ACG also plays a crucial role in cognitive and affective regulation (32). In previous studies, the reduction of FC between the right caudal dorsolateral area 6 and ACG in PD on state compared to PD off state was positively associated with improvements in rigidity and bradykinesia (8). In our study, early drug-naive PD patients with TD and PIGD subtypes have significantly lower cognitive scores and higher HAMD scores than HCs. TD patients showed increased connectivity between the left ACG and the A6CDL_R and the A4UL_R compared to PIGD patients. Multiple previous studies have shown that PD patients with PIGD type exhibit a quicker cognitive decline, a higher risk of depression indifference, a faster rate of disease development, and a lower quality of life than PD patients with TD type (3–5). Therefore, we speculate that TD patients achieve cognitive, movement, and emotional compensation by enhancing FC between the ACG and A6CDL_R and the A4UL_R during disease progression.

A key node that is crucial to the pathogenesis of PD is the basal ganglia. CAU and PUT belong to the basal ganglia (BG), whose functional abnormalities are considered to be the main cause of PD (33). Particularly, the basal ganglia maintain motor and sensory functioning through the regulation and integration of signals from the substantia nigra, thalamus, and cortex (34). As it is believed to hold long-term representations of object-related activities, the left SMG was considered a critical location for transitive praxis functions (35). The functional connectivity between the SMG and the M1, BG, and frontal regions was considerably stronger in PD patients in the ON state than in the OFF state (36). We revealed that the increased FC between A4UL and CAU, PUT, and left SMG may be crucial in the functional regulation of motor circuits in drug-naive PD patients with TD and PIGD subtypes.

Previous research showed a connection between the frontal and insula lobes and postural control (30), which may offer a sound foundation for the investigation of two motor subtypes in PD. The MFG and ORBinf were seen as important nodes in the motor inhibition network as parts of the prefrontal network, which may block the response to a discordant stimulus by suppressing the tendency for the motor system to automatically respond (37). In general, it is thought that the insula plays a key role in both cognitive and motor control. The insula is engaged in processing a variety of bodily sensory inputs and integrating them with the emotional and motivational environment. It is believed that doing so serves as the impetus for intentional movement (38). In this study, the TD patients showed increased connectivity between the A4UL_R and the left ORBinf/right INS and between the A6CDL_R and the right MFG compared with PIGD patients, and the FC strength was positively correlated with tremor and TD scores or negatively correlated with PIGD scores. Previous research has shown that decreased FC between the right insula and bilateral MFG and between the left insula and bilateral SMA is associated with gait impairment in PD patients (30). Therefore, MFG, INS, and ORBinf are considered to play a critical role in the movement disorders of early PD patients with TD and PIGD subtypes, the FC between the A4UL_R and the left ORBinf/right INS, and between the A6CDL_R and the right MFG can be used as biomarkers for distinguishing TD from PIGD.

A wide range of motor and non-motor symptoms, including pathological and compensatory responses, have been linked to changes in the cerebellum and its cortical connections in Parkinson’s disease (39). When compared to controls, cerebellar–whole brain and cerebellar–cerebellar connectivity was higher in Parkinson’s patients who were not taking medication, while it was lower in Parkinson’s patients who were on medication (40). Therefore, our finding of FC enhancement between A4UL and CRBL in drug-naïve PD patients with TD and PIGD subtypes is considered to be a compensatory pattern. The compensating effect might contribute to the preservation of largely normal motor and non-motor functions.

The PoCG is a crucial sensory brain area that is primarily in charge of integrating information from numerous somatosensory stimuli and achieving accurate object and external stimulus recognition. Additionally, it accepts projections of social collaboration and emotional expression. According to a meta-analysis, the left PoCG is crucial for the non-motor symptoms of PD and could be a viable target for treatment intervention (41). Our study found decreased FC between A4UL_L and PoCG in PD patients with TD and PIGD subtypes, suggesting a decreased information transmission ability in the sensorimotor network in PD patients with two motor subtypes. The primary symptoms of PD are visual dysfunction, including complex visual hallucinations, reduced visual acuity, and color discrimination (34). In PD patients, there is a recognized impairment in the interaction between the visual and motor networks (42), which is crucial for learning and controlling movements (43). In this study, abnormal connectivity between the upper limb area and the CUN and IOG in TD and PIGD patients may be related to adaptation processes as a consequence of altered motor function. In *de novo* PD patients without treatment, previous investigations have revealed decreased FC between the right PoCG, right PreCG, right precuneus, and the PUT (44), which is in accordance with our findings.

There were many limitations that should be noted. First, the study’s small sample may restrict the generalization of our findings to all Parkinson’s disease, but strict inclusion requirements reduced the diagnostic bias. To verify our findings, additional large-scale collaborative investigations are required. Second, although this study revealed changes in M1 subregions in *de novo* untreated PD patients, the present study remains cross-sectional in nature. In order to clarify the functional indicators along the course of PD and to determine whether these indicators can be useful markers to diagnose and track the advancement of motor subtypes, longitudinal research should be carried out by following the individuals. Third, there are undoubtedly additional approaches to subtype PD patients, for example, in accordance with various genetic characteristics and/or clinical presentations, when taking into account other literature on the pathogenesis of PD. However, the TD vs. PIGD subtype, being one of the earliest clinical subtypes defined by Jankovic et al., was the focus of our study. Future research is also necessary to comprehend the significance of FC differences across PD classifications other than TD and PIGD in the resting state.

## Conclusion

The present study shows that PD patients with TD and PIGD subtypes disrupt the cortical motor pathways in complex ways due to dopamine deficits. It induces a decreased FC between A4UL_L and PoCG_L and bilateral CUN, possibly responsible for the TD and PIGD patients’ akinesia. It also induces an abnormal pattern of increased FC between A4UL_L and CRBL, CAU_R, and PUT_L; and between A4UL_R and ACG_L, CRBL4_5_B, PUT_L, CAU_R, SMG_L, and MFG_L, which may reflect compensatory mechanisms in two motor subtypes. Importantly, TD patients occupied more resources in the MFG, ORBinf, INS, and ACG, which may be related to the slower progression of motor and non-motor symptoms and can be used as neural markers to distinguish them from PIGD patients.

## Data availability statement

The original contributions presented in the study are included in the article/supplementary material, further inquiries can be directed to the corresponding author.

## Ethics statement

The studies involving human participants were reviewed and approved by Medical Ethics Committee of the Affiliated Brain Hospital of Nanjing Medical University. The patients/participants provided their written informed consent to participate in this study.

## Author contributions

WL conceived and organized the research. LY, JX, YW, and GZ collected the data. QW analyzed the data and wrote the manuscript. MY and JX revised the manuscript. WL authorized the final version of the manuscript for publication. All authors contributed to the article and approved the submitted version.

## Funding

This study was supported by the National Natural Science Foundation of China (81571348, 81701671, and 81903589), the National Key Research and Development Program of China (2016YFC1306600 and 2017YFC1310302), the Postgraduate Research and Practice Innovation Program of Jiangsu Province (SJCX20_0497), and the Science and Technology Program of Jiangsu Province (BE2019611).

## Conflict of interest

The authors declare that the research was conducted in the absence of any commercial or financial relationships that could be construed as a potential conflict of interest.

## Publisher’s note

All claims expressed in this article are solely those of the authors and do not necessarily represent those of their affiliated organizations, or those of the publisher, the editors and the reviewers. Any product that may be evaluated in this article, or claim that may be made by its manufacturer, is not guaranteed or endorsed by the publisher.

## Footnotes

^1^ http://rfmri.org/dpabi

^2^ http://www.fil.ion.ucl.ac.uk/spm

^3^ http://atlas.brainnetome.org
